# Changes in Cortical Thickness Are Associated With Cognitive Ability in Postoperative School-Aged Children With Tetralogy of Fallot

**DOI:** 10.3389/fneur.2020.00691

**Published:** 2020-07-17

**Authors:** Siyu Ma, Yaping Li, Yuting Liu, Cheng Xu, Huijun Li, Qiong Yao, Ying Wang, Zhaocong Yang, Pengcheng Zuo, Ming Yang, Xuming Mo

**Affiliations:** ^1^Department of Cardiothoracic Surgery, Children's Hospital of Nanjing Medical University, Nanjing, China; ^2^Department of Radiology, Children's Hospital of Nanjing Medical University, Nanjing, China

**Keywords:** tetralogy of fallot, cerebral cortical thickness, cognition, VIQ, brain injury

## Abstract

In children with tetralogy of Fallot (TOF), there is a risk of brain injury even if intracardiac deformities are corrected. This population follow-up study aimed to identify the correlation between cerebral morphology changes and cognition in postoperative school-aged children with TOF. Resting-state functional magnetic resonance imaging (rs-fMRI) and the Wechsler Intelligence Scale for Children–Chinese revised edition (WISC-CR) were used to assess the difference between children with TOF and healthy children (HCs). Multiple linear regression showed that the TOF group had a lower verbal intelligence quotient (VIQ, 95.000 ± 13.433, *p* = 0.001) than the HC group and that VIQ had significant positive correlations with the cortical thickness of both the left precuneus (*p* < 0.05) and the right caudal middle frontal gyrus (*p* < 0.05) after adjustment for preoperative SpO2, preoperative systolic blood pressure (SBP), preoperative diastolic blood pressure (DBP) and time of aortic override (AO). Our results suggested that brain injury induced by TOF would exert lasting effects on cortical and cognitive development at least to school age. This study provides direct evidence of the relationship between cortical thickness and VIQ and of the need for strengthened verbal training in school-aged TOF patients after corrective surgery.

## Introduction

Tetralogy of Fallot (TOF) is a common cyanotic congenital heart disease (CHD) (1), accounting for almost 3.5% of CHD cases (2). TOF is mainly characterized by a ventricular septal defect (VSD), right ventricular outflow track obstruction, aortic override (AO) and right ventricular hypertrophy (3), all of which cause haemodynamic abnormalities that lead to a series of events including hypoxia episodes, brain abscesses, atrial fibrillation and cerebrovascular accidents (4). Under 1% of TOF patients who do not have surgery survive to 40 years old (5), whereas, those who undergo surgery before 5 years old have a 30-year survival rate of 90% (6). However, although mortality declines after surgery, survivors still face a variety of complications (7), such as myocardial damage (2), pulmonary incompetence (8), aortic dilation and arrhythmias (9). Furthermore, over 50% of TOF patients also exhibit cerebral damage (10), which may manifest as declines in cognitive, psychosocial, and behavioral ability (11, 12) and influence the patients' long-term quality of life (13).

Brain injury in TOF patients is an important concern (14). Children with TOF may suffer from brain injury in the prenatal (15), postpartum preoperative, perioperative (16), and postoperative periods due to genetic mutations, brain blood flow disturbances, cardiopulmonary bypass (CPB) (17, 18), anesthesia procedures (19), low cardiac output (LCO) (20) and socioeconomic status (SES) (21, 22). Of these time periods, the prenatal and postpartum preoperative periods are considered to carry the highest risk of brain injury (23, 24); additionally, they overlap with a critical period of cerebral development (25, 26). Fortunately, with advances in neuroimaging technology, brain injuries in children with CHDs are frequently identified early by magnetic resonance imaging (MRI) (27, 28); these brain injuries include delayed cerebral maturation (29), brain volume decline (30), white matter injury (WMI) (31), and stroke (32). In addition, delayed cortical development has been reported in CHD fetuses and neonates; the manifestations include delayed cortical folding, cortical depth asymmetry and reduced cortical thickness (25, 33). However, those studies describe only preoperative and short-term postoperative morphological changes. It remains for additional research to trace long-term cortical changes and further examine the association between cortical changes and cognition.

The effects of cortical changes on the cognitive ability of postoperative children with TOF remain unclear. Therefore, we examined the cerebral morphology of postoperative TOF patients via MRI, evaluated their cognitive abilities and further analyzed the correlation between them. Interestingly, the results showed that the cortical thickness values of both the left precuneus and the right caudal middle frontal cortex had significant positive correlations with verbal intelligence quotient (VIQ).

## Materials and Methods

### Subjects

From November 2015 to June 2016, 13 school-aged children with TOF were validated for participation, and informed consent was obtained from their legal guardians on their behalf. Ten of those children eventually completed resting-state functional magnetic resonance imaging (rs-fMRI) examination, and the data fulfilled the criteria for further analysis. Every participating child with TOF underwent corrected surgery in Nanjing Children's Hospital of Nanjing Medical University and had no known hereditary syndromes or central nervous system diseases, such as Down syndrome, cerebral tumors or craniocerebral trauma. The control group consisted of 13 healthy children (HCs) who were matched with the TOF group by age, gender, and education level and had no cardiovascular or nervous system diseases; informed consent was acquired from the children's legal guardians. All participants were right-handed and had no contraindications to MRI, such as implanted pacemakers or claustrophobia.

### Rs-fMRI Data Acquisition

MRI data were acquired from all participants using a Siemens MAGNETOM Avanto 1.5 T MRI machine with a standard 12-channel head coil. Subjects lay supine with sponge plugs in the external auditory canals to reduce the effect of scanner noise, and they were requested to lie awake quietly with their eyes closed and avoid thinking. When questioned afterward, all of the participants confirmed that they had not fallen asleep in the scanner. During the scans, each subject's head was braced with foam padding to reduce movement artifacts.

The scanning sequences are as follows: (1) *Fluid-attenuated inversion recovery (FLAIR):* the thickness was 5 mm, and there were no gaps between slices. The repetition time (TR) was 1,200 ms, and the echo time (TE) was 28 ms. The matrix size was 512^*^464. The total number of layers was 20. Intracranial lesions were identified and excluded by experienced radiologists. (2) *T1-weighted imaging (T1WI):* a three-dimensional magnetization prepared rapid gradient echo (3D-MP-RAGE) imaging sequence was used. TR was 1,900 ms, and TE was 2.48 ms. The turning angle was 9°. The field of view (FOV) was 256^*^256 mm, and the matrix size was 256^*^256. The protocol included 176 slices with a thickness of 1 mm each, and there were no gaps between slices. The T1WI scans were reconstructed into 3D images with a slice thickness of 4 mm and no space between slices. (3) *Resting blood oxygenation level-dependent (BOLD) scan:* gradient-echo echo-planar imaging (GRE-EPI) was applied. TR was 2,000 ms, and TE was 25 ms. The turning angle was 90°. The FOV was 240^*^240 mm, and the matrix size was 64^*^64. The slice thickness was 5 mm, with 2 mm spacing. The total number of slices was 36, and the scanning time was 6 min.

### Rs-fMRI Data Pre-processing

Data in this experiment were preprocessed with Data Processing Assistant for rs-fMRI (34) (DPARSF, http://www.restfmri.net) on the MATLAB platform.

The steps were as follows:

1. A total of 180 time points were collected by BOLD in this experiment. In order to reduce the influence of MRI magnetic field instability and noise during the initial scan, data from the first 10 time points were removed.
2. Slice-timing correction was carried out for the remaining time points so that all images collected at one time would be temporally aligned.
3. Correct head movements (including translation and rotation in 3D space). Considering the long scan time and the influence of magnetic resonance noise, some degree of head movement may occur, causing the haemodynamic response to be obscure. Therefore, it was necessary to correct the head movement of all subjects. Subjects were removed from this study if their head movement exceeded 1 mm of translation or 1° of rotation about the x, y, or z axis.
4. Spatial registration and linear detrending. Considering the differences in brain morphology among different subjects, all MRI images were standardized to the same reference space (standard anatomical template for the head, Montreal Neurological Institute, Canada), and the standardized data were then processed to remove linear trends.
5. Low-frequency filtering. A frequency band of 0.01–0.08 Hz was used to filter out low-frequency drift.
6. Spatial smoothing. A Gaussian kernel function with a full width at half maximum (FWHM) of 4 × 4 × 4 mm^3^ was used to perform spatial smoothing of fMRI images.

### Cerebral Morphology Analyses

We used the Computational Anatomy Toolbox (CAT12, http://dbm.neuro.uni-jena.de/cat/) of SPM12 (https://www.fil.ion.ucl.ac.uk/spm/software/spm12/) to extract morphological indexes of the cortical surface. An image of each subject was produced in a standard position, such that every image had the same origin and spatial direction. Linear and non-linear registration of high-resolution T1WI was performed for each patient. The image was divided into gray matter volume (GMV), white matter volume (WMV) and cerebrospinal fluid volume (CSFV). Brain tissue volume (BTV) and total intracranial volume (TIV) could then be calculated.

### Intelligence Assessment

All subjects had their cognitive ability assessed using the Wechsler Intelligence Scale for Children–Chinese revised edition (WISC-CR). The WISC is an authoritative and widely used intelligence scale for children (12). The WISC-CR was suitable for Chinese children between the ages of 6 and 16 years; the test is divided into 12 domains, including common sense, analogies, arithmetic, vocabulary, comprehension, digit span, missing picture completion, picture arrangement, block design, object collocation, decoding, and mazes (of these, digit span and mazes were optional). Each subject's results were scored according to the operating manual and the subject's age. The VIQ was determined from the first six items, and the performance intelligence quotient (PIQ) was determined from the last six items. Finally, the full-scale intelligence quotient (FSIQ) could be calculated.

### Statistical Analyses

We used SPSS 20.0 (IBM Corp., Armonk, NY, USA) to perform the statistical analyses in this study. Continuous variable data are described as the mean ± SD in Tables 1, 2. Differences between the TOF group and the HC group were shown in some variables by conducting one-sample *t*-tests. Single and multiple linear regression analyses were used to explore the correlation between cortical average thickness of brain regions, intelligence quotient (IQ) and covariates. Statistical significance was considered when the *p* < 0.05.

**Table 1:** Characteristics of TOF and healthy children.

**Variables**	**TOF**	**HC**	***p*-value**
	**(*n* = 10)**	**(*n* = 13)**	
Age (year)	10.01 ± 1.88	9.73 ± 0.77	0.63
Sex (male/female)	6/4	8/5	0.94
Education (year)	2.31 ± 1.25	2.42 ± 0.91	0.80
Household income (Yuan per month)	6,750.00 ± 2,214.22	7,192.31 ± 1,575.05	0.58
Age of surgery (year)	2.10 ± 1.69	NA	
Postoperative time (year)	7.33 ± 2.31	NA	
Hospital stays (day)	17.38 ± 5.55	NA	
Preoperative SpO_2_ (%)	73.29 ± 16.11	NA	
Preoperative SBP (mmHg)	98.57 ± 11.63	NA	
Preoperative DBP (mmHg)	57.57 ± 9.74	NA	
Preoperative pH	7.34 ± 0.03	NA	
CPB time (min)	61.75 ± 7.52	NA	
AO time (min)	38.52 ± 5.38	NA	

*Mean ± SD. TOF, tetralogy of Fallot; HC, healthy children; SpO_2_, saturation of pulse oxygen; SBP, systolic blood pressure; DBP, diastolic blood pressure; pH, potential of hydrogen; CPB, cardiopulmonary bypass; AO, aortic occlusion; NA, not available. Bold values represents that the results have statistical significance*.

**Table 2:** Cerebral morphology and intelligence quotient changings in postoperative TOF.

		**TOF**	**HC**	***p*-value**
CSFV		246.200 ± 53.425	266.769 ± 41.698	0.155
GMV		683.800 ± 75.295	749.692 ± 42.092	**0.007**
WMV		438.000 ± 56.978	496.308 ± 48.340	**0.007**
BTV		1,121.800 ± 129.049	1246.000 ± 86.085	**0.006**
TIV		1,368.100 ± 151.542	1513.154 ± 111.126	**0.007**
Whole brain CAT		2.750 ± 0.061	2.842 ± 0.068	**0.001**
**Left hemisphere CAT**				
	Inferior temporal	2.881 ± 0.138	3.000 ± 0.149	**0.032**
	Lateral occipital	2.016 ± 0.111	2.123 ± 0.079	**0.007**
	Middle temporal	3.182 ± 0.089	3.236 ± 0.145	0.156
	Fusiform	2.732 ± 0.135	2.919 ± 0.153	**0.003**
	Isthmus cingulate	2.623 ± 0.266	2.656 ± 0.199	0.368
	Precuneus	2.671 ± 0.148	2.799 ± 0.076	**0.007**
**Right hemisphere CAT**				
	Superior frontal	3.286 ± 0.103	3.346 ± 0.309	0.282
	Caudal middle frontal	2.959 ± 0.141	3.088 ± 0.101	**0.009**
VIQ		95.000 ± 13.433	122.000 ± 9.138	**0.001**
PIQ		98.600 ± 18.014	104.200 ± 12.755	0.274
FSIQ		96.100 ± 15.286	115.400 ± 10.213	**0.013**

*Mean ± SD. TOF, tetralogy of Fallot; HC, healthy children; CSFV, cerebrospinal fluid volume; GMV, gray matter volume; WMV, white matter volume; BTV, brain tissue volume; TIV, total intracranial volume; CAT, cortical average thickness; VIQ, verbal intelligence quotient; PIQ, performance intelligence quotient; FSIQ, full scale intelligence quotient. Bold values represents that the results have statistical significance*.

## Results

Table 1 shows the demography of TOF and HC groups. No significant difference was found in age, gender, years of education, or household income. Additionally, the hospital records of the TOF group are summarized.

**Figure 1:**
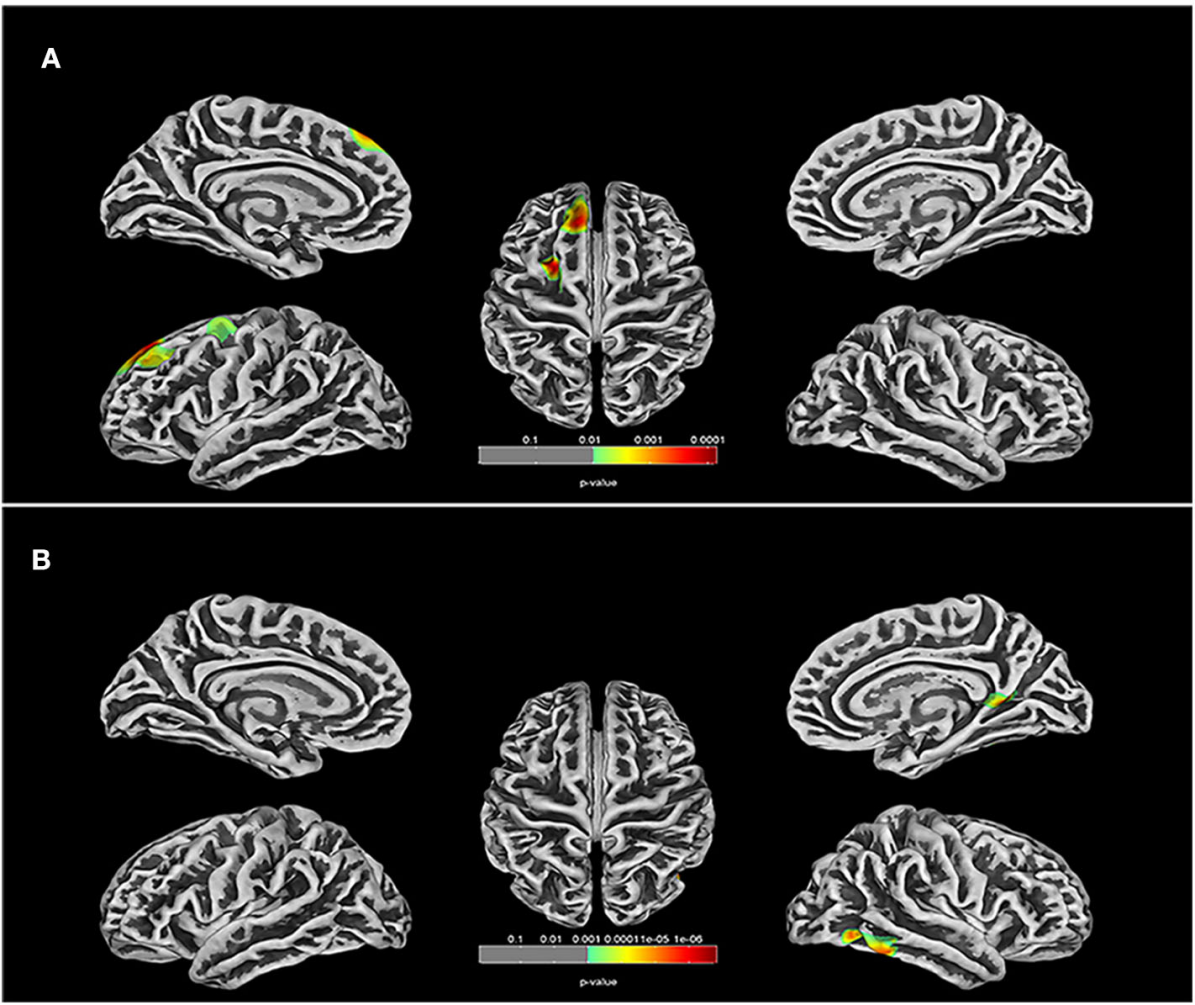
Comparison of cortical thickness in TOF and HC groups. **(A)** In left hemisphere of TOF group, the cortical thickness of inferior temporal, lateral occipital, middle temporal, fusiform, isthmus cingulate, and precuneus were reduced. **(B)** TOF children had lower cortical thickness of superior frontal and caudal middle frontal in right hemisphere.

Differences in cerebral morphology and IQ between the TOF and HC groups are shown in Table 2. Healthy children had higher VIQ (95.000 ± 13.433) and FSIQ (95.000 ± 13.433) scores than children with TOF. GMV (683.800 ± 75.295), WMV (438.000 ± 56.978), BTV (1,121.800 ± 129.049), TIV (1,368.100 ± 151.542) and whole-brain cortical average thickness (CAT, 2.750 ± 0.061) were reduced in children with TOF compared with healthy children. Furthermore, Figure 1 shows a 3D simulation diagram of differences in cortical thickness between the two groups. Reduced left inferior temporal CAT (2.881 ± 0.138), left lateral occipital CAT (2.016 ± 0.111), left precuneus CAT (2.671 ± 0.148) and right caudal middle frontal CAT (2.959 ± 0.141) were evident in the TOF group.

After analyzing the correlation among cerebral morphology changes, IQ and hospital records (Tables 3, 4), we found that preoperative SpO_2_, preoperative systolic blood pressure (SBP), preoperative diastolic blood pressure (DBP) and time of AO were related to morphology changes.

**Table 3:** Pearson correlation between cerebral structure changings and cognitive abilities.

	**VIQ**	**PIQ**	**FSIQ**
CSFV	0.058	−0.345	−0.202
GMV	0.453	0.687^*^	0.633^*^
WMV	0.392	0.464	0.457
BTV	0.438	0.605	0.571
TIV	0.395	0.395	0.416
Whole brain CAT	−0.296	−0.058	−0.141
Left inferior temporal CAT	0.511	0.576	0.627
Left lateral occipital CAT	−0.911^**^	−0.480	−0.749^*^
Left middle temporal CAT	−0.388	−0.556	−0.480
Left fusiform CAT	0.219	−0.213	−0.023
Left isthmus cingulate CAT	−0.032	0.097	0.035
Left precuneus CAT	0.080	0.283	0.271
Right superior frontal CAT	−0.321	−0.089	−0.182
Right caudal middle frontal CAT	0.060	0.119	0.143

* Correlation is significant at the 0.05 level,

** *Correlation is significant at the 0.01 level. CSFV, cerebrospinal fluid volume; GMV, gray matter volume; WMV, white matter volume; BTV, brain tissue volume; TIV, total intracranial volume; CAT, cortical average thickness; VIQ, verbal intelligence quotient; PIQ, performance intelligence quotient; FSIQ, full scale intelligence quotient. Bold values represents that the results have statistical significance*.

**Table 4:** Pearson correlation between cerebral structure changings and demographic variables.

	**Age**	**Hospital stays**	**Age of surgery**	**Postoperative time**	**Preoperative SpO_**2**_**	**Preoperative SBP**	**Preoperative DBP**	**Preoperative pH**	**CPB time**	**AO time**
CSFV	0.365	0.322	0.771^*^	−0.346	0.653	0.206	0.350	0.103	−0.098	−0.592
GMV	−0.446	−0.462	0.283	−0.534	0.704	0.259	0.006	0.168	−0.190	−0.208
WMV	−0.228	−0.282	0.320	−0.410	0.633	0.245	−0.235	−0.138	−0.201	−0.053
BTV	−0.363	−0.396	0.309	−0.496	0.695	0.261	−0.102	0.036	−0.201	−0.145
TIV	−0.109	−0.160	0.635	−0.572	0.887^**^	0.313	0.093	0.081	−0.211	−0.413
Whole brain CAT	−0.446	0.258	−0.213	−0.147	−0.146	0.018	0.585	0.584	−0.028	−0.372
Left inferior temporal CAT	0.138	−0.729	−0.553	0.528	−0.124	−0.070	−0.179	0.350	0.568	0.241
Left lateral occipital CAT	−0.003	0.567	0.155	−0.123	−0.344	−0.012	−0.134	−0.722	−0.383	0.285
Left middle temporal CAT	0.375	0.473	0.156	0.141	−0.178	−0.537	0.126	0.579	−0.049	−0.486
Left fusiform CAT	0.092	−0.184	−0.066	0.116	0.083	0.764^*^	0.816^*^	−0.165	0.526	0.033
Left isthmus cingulate CAT	0.074	−0.190	0.708	−0.500	0.929^**^	0.198	0.471	0.598	−0.032	−0.779^*^
Left precuneus CAT	−0.174	−0.288	−0.310	0.119	−0.143	−0.072	0.428	0.632	0.260	−0.197
Right superior frontal CAT	−0.227	0.272	0.082	−0.224	0.143	0.467	0.881^**^	0.279	0.118	−0.409
Right caudal middle frontal CAT	−0.388	0.115	−0.135	−0.167	0.123	0.250	0.742	0.671	0.140	−0.486

* Correlation is significant at the 0.05 level.

** *Correlation is significant at the 0.01 level. CSFV, cerebrospinal fluid volume; GMV, gray matter volume; WMV, white matter volume; BTV, brain tissue volume; TIV, total intracranial volume; CAT, cortical average thickness; SpO_2_, saturation of pulse oxygen; SBP, systolic blood pressure; DBP, diastolic blood pressure; pH, potential of hydrogen; CPB, cardiopulmonary bypass; AO, aortic occlusion. Bold values represents that the results have statistical significance*.

In multiple linear regression, left precuneus CAT (beta: 79.905; 95% CI: 72.226,87.584) and right caudal middle frontal CAT (beta: 143.606; 95% CI: 25.181,262.030) were related to VIQ after adjusting for preoperative SpO_2_, preoperative SBP, preoperative DBP and time of AO (Table 5).

**Table 5:** Multivariable association of cerebral structure changings and cognitive abilities in TOF postoperative children.

	**VIQ**		**FSIQ**	
	**Beta (95 % CI)**	***p*-value**	**Beta (95%CI)**	***p*-value**
CSFV	−0.167 (−1.681, 1.348)	0.396	−0.283 (−1.570, 1.004)	0.219
GMV	0.199 (−2.576, 2.974)	0.529	0.369 (−2.531, 3.269)	0.353
WMV	0.183 (−11.191, 11.556)	0.872	0.618 (−14.510, 15.746)	0.695
BTV	0.131 (−2.257, 2.520)	0.612	0.261 (−2.439, 2.962)	0.435
TIV	−0.545 (−3.892, 2.801)	0.286	−0.664 (−8.180, 6.852)	0.463
Whole brain CAT	290.098 (−1,670.986, 2,251.182)	0.311	471.829 (−813.154, 1,756.813)	0.134
Left inferior temporal CAT	51.623 (−233.107, 336.353)	0.261	63.877 (−602.372, 730.127)	0.438
Left lateral occipital CAT	−104.201 (−694.515, 486.113)	0.267	−128.442 (−1495.279, 1238.394)	0.444
Left middle temporal CAT	−5.064 (−2,135.642, 2,125.515)	0.981	−74.679 (−3057.549, 2908.192)	0.804
Left fusiform CAT	−288.834 (−1,961.253, 1,383.584)	0.272	−460.896 (−1,344.694, 422.902)	0.095
Left isthmus cingulate CAT	36.404 (−1,271.914, 1,344.721)	0.784	9.942 (−2,023.929, 2,043.813)	0.961
Left precuneus CAT	**79.905 (72.226, 87.584)**	**0.005**	112.598 (−307.122, 532.318)	0.182
Right superior frontal CAT	−733.161 (−7,193.769, 5,727.447)	0.386	−830.596 (−13,705.810, 12,044.617)	0.563
Right caudal middle frontal CAT	**143.606 (25.181, 262.030)**	**0.041**	206.552 (−361.043, 774.147)	0.136

*Adjusted for preoperative SpO_2_, preoperative SBP, preoperative DBP and AO time. CSFV, cerebrospinal fluid volume; GMV, gray matter volume; WMV, white matter volume; BTV, brain tissue volume; TIV, total intracranial volume; CAT, cortical average thickness; SpO_2_, saturation of pulse oxygen; SBP, systolic blood pressure; DBP, diastolic blood pressure; AO, aortic occlusion; VIQ, verbal intelligence quotient; FSIQ, full scale intelligence quotient; CI, confidence interval. Bold values represents that the results have statistical significance*.

## Discussion

Our population follow-up study was the first to identify the relationship between reduced cortical thickness and low VIQ in postoperative school-aged children with TOF, especially in the precuneus of the left hemisphere and caudal middle frontal cortex of the right hemisphere.

The cerebral cortex plays an irreplaceable role in interconnecting brain areas and in sensory, motor and cognitive processing (35) and should be given close attention in postoperative children with TOF. Children with TOF have reduced oxygen delivery and oxygen consumption (36), to which the cerebral cortex is especially vulnerable (25). The main microstructural changes that occur in the hypoxic cerebral are impaired dendritic arborization of neurons (37) and inhibition of glial cell formation (35, 38, 39). Although hypoxia induced by CHD influences dendritic outgrowth, cortical connectivity, and synapse formation (40, 41), few studies have explored the association between cortical alterations and cognition in postoperative children with TOF. Thus, based on our results and published studies, we speculate herein about the underlying mechanism of low VIQ induced by reduced cortical thickness in postoperative school-aged children with TOF.

The precuneus might influence verbal cognition by combining the temporal lobes via the temporoparietal junction (TPJ). The precuneus, part of the posteromedial parietal cortex, is part of the associative cortices (42). The precuneus may be involved in visual-spatial imagery processing, episodic memory retrieval and self-processing operations by mutually connecting with the frontal lobe, dorsal premotor area, supplementary motor area, anterior cingulate cortex, and temporoparietooccipital cortex (TPO) (42–44). In addition, many studies have indicated that the precuneus is associated with verbal processing (45–47). However, few studies have examined the mechanism by which the precuneus influences verbal cognition. Recently, studies showed that the precuneus and temporal cortex could participate in attention, social cognition and working memory by interconnecting via short U-shaped fibers and long connections (48, 49), which was contrary to the traditional notion in neuropsychology that the precuneus and temporal cortex function as separate regions (50). Furthermore, the left TPJ showed hyperactivity in patients with auditory verbal hallucinations (AVHs) (49), and the left temporal lobe is related to the language development of children with CHD (51). Thus, we hypothesized that decreased left precuneus cortical thickness could reduce the connection of the temporal lobe via the TPJ and further influence VIQ in postoperative children with TOF.

The middle frontal lobe is known as the secondary language area (52, 53), and it is involved in many verbal expression processes, such as verbal and non-verbal fluency (54–56), verbal working memory (57–59), switching (60), and semantics (61). The right frontal lobe mainly participates in orthography of Chinese characters, verbal suppression, verbal strategy use and non-verbal fluency (54, 62, 63). Our results also provided valid proof that the middle frontal cortex is correlated with verbal cognition. Therefore, we infer that children with TOF had poor performance in verbal expression and led to lower VIQ.

However, our study has some weaknesses. First, a larger sample size would be necessary to make the results more credible. Moreover, this study merely evaluated the overall neurological function of school-aged children after TOF correction and could not identify the specific timing of injuries due to the absence of preoperative MRI results. In addition, it is mainly children with neurological disorders who tend to participate in these programmes, and the results might be biased accordingly. Furthermore, this study reported a correlation between cortical thickness and IQ. The influence of other morphological changes on cognition is expected to be shown in future articles. Finally, this study was based on the preliminary exploration of TOF cases, and functional validation is needed to explore the underlying mechanism.

## Conclusion

In this study, the VIQ of postoperative school-aged children with TOF was lower than that of healthy children of similar age and was correlated with reduced cortical thickness of the precuneus in the left hemisphere and the caudal middle frontal cortex in the right hemisphere. The results implied that brain damage before the correction of intracardiac deformity continuously influenced the cortical development and cognitive abilities of children with TOF, at least to school age. The underlying mechanisms might consist of delayed language development affected by left precuneus-temporal connections and poor verbal expression induced by right middle frontal cortex dysfunction. Therefore, even if the intracardiac deformities of TOF patients are corrected, the possibility of brain injury should also be given close attention after surgery. Furthermore, language expression training for children with TOF should be strengthened after corrective surgery.

## Data Availability Statement

The original contributions presented in the study are included in the article/supplementary material, further inquiries can be directed to the corresponding author/s.

## Ethics Statement

The studies involving human participants were reviewed and approved by the Ethics Committee of Children's hospital of Nanjing Medical University. Written informed consent to participate in this study was provided by the participants' legal guardian/next of kin. Written informed consent was obtained from the individual(s), and minor(s)' legal guardian/next of kin, for the publication of any potentially identifiable images or data included in this article.

## Author Contributions

XM and MY designed this study protocol. YLi collected the information of population. HL, QY, and YW scanned the participants by MRI. CX, YLiu, and SM performed the data analysis under the close supervision of ZY, PZ, MY, and XM. The manuscript was drafted by SM and proofread by CX. All authors contributed to the article and approved the submitted version.

## Conflict of Interest

The authors declare that the research was conducted in the absence of any commercial or financial relationships that could be construed as a potential conflict of interest.
